# Role of the lateral prefrontal cortex in speech monitoring

**DOI:** 10.3389/fnhum.2013.00703

**Published:** 2013-10-29

**Authors:** Stephanie K. Riès, Kira Xie, Kathleen Y. Haaland, Nina F. Dronkers, Robert T. Knight

**Affiliations:** ^1^Department of Psychology, Helen Wills Neuroscience Institute, University of CaliforniaBerkeley, CA, USA; ^2^New Mexico Veterans Affairs Healthcare System, University of New MexicoAlbuquerque, NM, USA; ^3^Department of Psychiatry, University of New MexicoAlbuquerque, NM, USA; ^4^Department of Neurology, University of New MexicoAlbuquerque, NM, USA; ^5^Veterans Affairs Northern California Health Care System and University of CaliforniaDavis, CA, USA

**Keywords:** on-line speech monitoring, prefrontal lesions, error negativity, electroencephalography, brain networks, overt picture-naming

## Abstract

The role of lateral prefrontal cortex (LPFC) in speech monitoring has not been delineated. Recent work suggests that medial frontal cortex (MFC) is involved in overt speech monitoring initiated before auditory feedback. This mechanism is reflected in an event-related potential (ERP), the error negativity (Ne), peaking within 100 ms after vocal-onset. Critically, in healthy individuals the Ne is sensitive to the accuracy of the response; it is larger for error than correct trials. By contrast, patients with LPFC damage are impaired in non-verbal monitoring tasks showing no amplitude difference between the Ne measured in correct vs. error trials. Interactions between the LPFC and the MFC are assumed to play a necessary role for normal action monitoring. We investigated whether the LPFC was involved in speech monitoring to the same extent as in non-linguistic actions by comparing performance and EEG activity in patients with LPFC damage and in aged-matched controls performing linguistic (Picture Naming) and non-linguistic (Simon) tasks. Controls did not produce enough errors to allow the comparison of the Ne or other ERP in error vs. correct trials. PFC patients had worse performance than controls in both tasks, but their Ne was larger for error than correct trials only in Naming. This task-dependent pattern can be explained by LPFC-dependent working-memory requirements present in non-linguistic tasks used to study action monitoring but absent in picture naming. This suggests that LPFC may not be necessary for speech monitoring as assessed by simple picture naming. In addition, bilateral temporal cortex activity starting before and peaking around vocal-onset was observed in LPFC and control groups in both tasks but was larger for error than correct trials only in Naming, suggesting the temporal cortex is associated with on-line monitoring of speech specifically when access to lexical representations is necessary.

## INTRODUCTION

Monitoring our speech production on-line in order to produce intelligible utterances is key to effective communication. The last four decades of psycholinguistic research has led to various theories of how speech monitoring is performed. These theories generally distinguish our ability to monitor speech *before* vs. *after* it is overtly produced. When speech production is monitored on-line, before it is produced, the process is referred to as the “inner loop” of speech monitoring. After speech has been produced, monitoring is referred to as the “outer loop” as it relies primarily on external auditory feedback (for a review, see Postma, 2000). However, theories of speech monitoring differ notably in the types of representations involved. While some theories propose that speech monitoring relies almost exclusively on the language comprehension system (e.g., the “perceptual loop” theory, Levelt, 1983, 1989), others suggest monitoring takes place within the language production system itself (e.g., “production-based” accounts, e.g., Laver, 1973, 1980; Schlenck et al., 1987). Despite these differences, one common implicit assumption has been that speech monitoring relies on mechanisms that are inherent to the language system. More recently, this assumption has been challenged (for a detailed theoretical account, see Nozari et al., 2011).

Functional imaging studies of speech monitoring have pointed to brain activity specifically associated with speech monitoring, but also to others common to speech and general action monitoring. Using tasks in which verbal auditory feedback is manipulated, functional magnetic resonance imaging (fMRI) and positron emission tomography (PET) studies suggest temporal regions, and especially the posterior superior temporal gyrus (pSTG), but also medial frontal regions, including the anterior cingulate cortex (ACC) and the supplementary motor area (SMA), are associated with speech monitoring (for a meta-analysis, see Indefrey, 2011). On the one hand, bilateral temporal lobe activations have been specifically associated with speech monitoring and seem more precisely tied to auditory feedback (i.e., after speech has been produced). On the other hand, medial frontal regions have been repeatedly associated with general action monitoring as well (for review, see Ridderinkhof et al., 2004a) and thus have been hypothesized to host a domain-general monitoring process (Barch et al., 2000; Christoffels et al., 2007a). Comparing auditory feedback distortion to normal speech, Van de Ven et al. (2009) investigated functional connectivity between temporal and medial frontal regions. Their results suggest that the activations of these brain regions are inversely related. Stronger activation of the ACC and SMA but reduced activation of the STG under normal verbal feedback condition is associated with increased monitoring difficulty. However, because of limited temporal resolution, these studies have not been able to disentangle the specific roles of these brain regions in speech monitoring. Thus, the network of brain regions engaged in “inner” vs. “outer” speech monitoring or both is not defined.

Electroencephalographic (EEG) investigations examining the “error negativity” (Ne) have reinforced the idea that speech monitoring relies partly on a monitoring system common to speech and other actions. The Ne is an event-related negative potential initially observed following the onset of erroneous responses in non-linguistic tasks (Falkenstein et al., 1991; Gehring et al., 1993) and more recently also in linguistic tasks involving overt speech production (Masaki et al., 2001). The Ne peaks shortly after the beginning of the vocal response (around 70 ms after vocal onset), has a fronto-central scalp distribution with proposed sources in the ACC and/or SMA (e.g., Dehaene et al., 1994; Debener et al., 2005). Importantly, a similar albeit smaller potential of presumably similar origin (Roger et al., 2010) was later reported in correct trials in non-linguistic tasks (Vidal et al., 2000, 2003; Bartholow et al., 2005) and also recently in overt speech production (Riès et al., 2011; Acheson et al., 2012). This suggested the Ne does not reflect error detection per se but rather a general-purpose response monitoring system associated with, but independent of, error detection. Methodological difficulties linked to articulatory electromyographic activity in the EEG signal had previously prevented the study of the Ne in correct utterances. These were overcome using a blind-source separation algorithm based on canonical correlation analysis (BSS-CCA, De Clercq et al., 2006; De Vos et al., 2010). Critically, recent work has revealed the Ne in errors and in correct trials emerges before vocal onset (Riès et al., 2011); it starts to rise before auditory feedback can be perceived. This supports the notion that a general-purpose action monitoring mechanism hosted in the medial frontal lobe subserves inner speech monitoring.

In sum, the proposed involvement of a domain-general monitoring system in inner speech monitoring is supported by modulation of the BOLD signal in the ACC in situations in which the constraints on speech monitoring are manipulated; the observation of the Ne in overt speech and; the observations that the Ne is modulated similarly by time-pressure, competition between representations or uncertainty of the response (Ganushchak and Schiller, 2006, 2008a, b, 2009; see also Nozari et al., 2011; Acheson et al., 2012, for a neuropsychological and computational investigation of this domain-general account). In other actions however, the mechanism reflected by the Ne has been shown to be dependent not only on medial frontal regions such as the ACC but also on other cortical and subcortical regions, suggesting action monitoring is dependent on a network of interacting brain regions. Investigation of patients with lateral prefrontal cortex (LPFC) damage due to stroke using a non-verbal task (i.e., a variant of the Flanker task), Gehring and Knight (2000) suggested the monitoring mechanism reflected by the Ne is dependent on the integrity of the LPFC and not solely on medial frontal regions. Indeed, these PFC patients did not show an amplitude difference between the Ne measured in correct vs. incorrect trials, indicating interactions between the LPFC and the medial frontal region are necessary for normal action monitoring. This result was replicated by Ullsperger et al. (2002). These authors also tested patients with orbitofrontal cortex (OFC) damage and others with temporal cortex damage. Only the patients with LPFC damage showed no amplitude difference between the Ne measured on error vs. correct trials (although, see Turken and Swick, 2008, for a possible involvement of the OFC). Finally, the basal ganglia, the thalamus, and prefrontal and motor cortico-striato-thalamo-cortical circuits have also been associated with action monitoring as shown in patients with Parkinson’s disease (Falkenstein et al., 2001), in patients with basal ganglia and white matter lesions (Ullsperger and von Cramon, 2006), and in patients with thalamic lesions (Peterburs et al., 2011) who also show a reduced Ne compared to controls. In sum, numerous studies of action monitoring indicate the domain-general mechanism reflected by the Ne is influenced by other brain regions in addition to the ACC, including the LPFC which is the focus of the current study. Importantly, these regions appear to play complementary but different roles in the cognitive control of goal-directed behavior (see Ridderinkhof et al., 2004a for a detailed review). Indeed, whereas the ACC seems to be the core region for on-line action monitoring, the LPFC seems more particularly involved in implementing corrective behavior (Gehring and Knight, 2000; Ridderinkhof et al., 2004b). Finally and critically for the present study, the LPFC has been shown to play a role in maintaining arbitrary associations between visual cues and actions in an active state until the goal is reached, especially in situations in which there is interference between possible responses (for a review, see Miller and Cohen, 2001).

In the language domain, production studies investigating interactions between brain regions subserving on-line speech monitoring are scarce. The limited temporal or spatial resolution of the available imaging techniques and the problem posed by articulation-related artifacts in EEG and MEG have contributed to the paucity of research in this area. Thus, whether or not the domain-general monitoring process involved in inner speech monitoring and indexed by the Ne is dependent on the LPFC has not been directly investigated.

Patients with lesions to the LPFC have language production deficits characterized by impaired verbal fluency which varies depending on the specific area of PFC damage and the extent of the lesion (Goodglass, 1993). Whereas lesions restricted to Broca’s area (BA44/45) have been shown to cause transient mutism resolving in 3–6 weeks to an anomic aphasia (Mohr et al., 1978), much greater dysfunction can be caused if the lesion encompasses underlying white matter pathways and adjacent cortical structures producing Broca’s aphasia (Dronkers et al., 2007). Patients with lesions to the left PFC and neighboring regions can therefore make various types of errors depending on the lesion location and extent. However, the LPFC has generally not been associated with speech monitoring in studies focused on this process [see meta-analyses by Indefrey and Levelt (2004), Indefrey (2011), and a detailed review by Price (2012)]. This suggests the domain-general monitoring system involved in inner speech monitoring may not be as dependent on LPFC as in non-speech monitoring. We hypothesize that the reason for this may be the nature of the stimulus-response associations involved. In the tasks used to study general action monitoring, stimulus-response associations are often arbitrary and thus need to be maintained in working memory (e.g., the Flanker task, Eriksen and Eriksen, 1974). In the tasks used to study speech monitoring (e.g., simple picture naming in which auditory feedback is masked or not; or verb generation), arbitrary rules do not need to be maintained in PFC dependent working memory as the relation between the stimulus and the response is rooted in long-term memory. We propose the general purpose monitoring system subserving inner speech monitoring as assessed by simple picture naming relies on a network of brain regions partly independent from the one subserving other actions probed with tasks involving arbitrary stimulus-response associations.

To address this hypothesis we recorded EEG in a cohort of patients with lesions centered in the left or right PFC and in aged-matched controls as they performed a simple overt picture naming task and a verbal Simon task. The Simon task (Craft and Simon, 1970) involves an arbitrary relationship between the stimulus and the response based on a rule determined by the experimenter. It has been used extensively in the study of non-linguistic cognitive control (e.g., see Lu and Proctor, 1995 for a review) and has also been used with verbal instead of manual responses (Proctor and Vu, 2002; Wühr, 2006). Here we used a verbal version so that output processes would be comparable in both tasks. We combine spatial (PFC lesion) and temporal information to inform the network of regions involved in speech monitoring before auditory feedback can be perceived. We addressed the problem posed by articulation-related artifacts as described in Riès et al. (2011), 2013, enabling us to observe clear components peaking around and after vocal onset. Our main hypothesis is that interactions between medial-frontal regions and the LPFC are not as critical for on-line speech monitoring as assessed by picture naming as they are for non-linguistic actions as assessed by tasks involving arbitrary stimulus-response associations. We therefore predict an amplitude difference between the Ne in incorrect vs. correct trials in Naming but not in the Simon task. More specifically, the Ne should be larger in incorrect than in correct trials in Naming but not in the Simon task. In addition, we also examined the timing of error-related event-related potentials (ERP) recorded over the temporal lobes for two reasons. First we aimed to assess whether or not temporal cortex is involved in inner speech monitoring or is limited to outer speech monitoring. Based on the results described in correct trials in overt picture-naming (Riès et al., 2011), we predicted larger temporal activity in incorrect vs. correct trials starting before auditory feedback, suggesting underlying mechanisms linked not only to outer speech monitoring but also to inner speech monitoring. Second, we wanted to assess whether the nature of the response selection process (linguistic or not) had an impact on the involvement of temporal cortices in action monitoring. Responses in both tasks involves overt speech but Naming requires access to lexical representations to a much greater extent than the Simon task. We predicted the temporal cortex would be involved in speech monitoring as assessed by the naming task but to a lesser degree in the Simon task. Specifically, larger temporal activities should be observed in error vs. correct trials in Naming but not in the Simon task.

## MATERIALS AND METHODS

### PARTICIPANTS

A total of 17 patients (7 males; mean age: 63.9, SD = 12.6 years old) with focal unilateral lateral frontal lesions (6 right, 11 left hemisphere) were recruited to participate in the study. All right frontal patients had no language impairment as diagnosed by neurological assessment. All left frontal patients were examined on at least two subtests of the Western Aphasia Battery (WAB; Kertesz, 1982), measuring spontaneous speech (assessing general conversational speech production abilities; maximum score of 20), and comprehension of sequential commands (assessing general speech comprehension skills; maximum score of 80). We note the score of one patient on Sequential Commands was not available; we only had the overall WAB comprehension score (grouping three comprehension subtests including the Sequential Commands).

Three left frontal patients (two males) could not perform the experimental tasks adequately due to marked aphasia: they either did not understand the instructions properly or their error rate on the experimental tasks was over 40% (mean score on Sequential Commands: 73/80, SD = 8.19, individual scores: 64, 75, and 80; mean score for Spontaneous Speech: 17/20, SD = 2.64, individual scores: 18, 19, and 14, respectively; thus the one patient who had a good comprehension score of 80 had a poor production score of 14). EEG could not be recorded in two other left PFC patients (two females): one could not sit for the entire duration of the experiment and EEG recording had to be interrupted and the other had a sore spot on the scalp that bothered her. The data of these five left PFC patients were excluded from the analysis.

**FIGURE 1:**
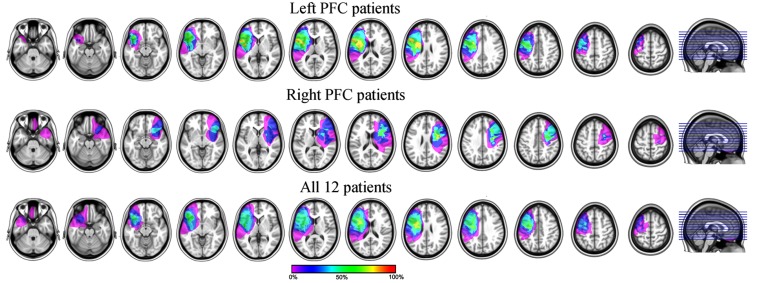
**Lesion overlapping of the six left (top) and six right (middle) and all (bottom) PFC patients included in the analyses.** For the bottom row, the lesions of the right PFC patients were mirrored on the left side of the brain.

The remaining six left PFC patients had a mean Spontaneous Speech score of 19/20 (SD = 0.63), reflecting overall good production abilities despite some articulation problems (one patient had a score of 18 reflecting a lack of detail in the picture description or in answering one of the questions). The mean Sequential Command score was of 76/80 (SD = 9.17; this average was made for the five patients for which we had the Sequential Command score. The last patient had an overall Comprehension score of 9.8/10 reflecting good comprehension abilities). We note that four out of the five patients had a perfect score of 80, only one had a relatively low score of 59.5. This patient asked to be reminded of the Simon task rule regularly at the breaks but was nevertheless able to perform both tasks correctly. Thus the language production deficits of the left PFC group we kept for further analysis were overall mild in nature allowing the patients to perform the tasks adequately. Lesion overlapping of the 12 remaining patients is presented in **Figure 1**.

The data of 12 controls (5 males; mean age: 62, SD = 11.6 years old) matched in age, gender, and education to the remaining 12 patients [5 males; mean age: 61.2, SD = 10.8 years old, *t*(21.90) < 1] were collected. Patients had on average 16.6 years of education (SD = 3.0) and controls had on average 16.7 years of education [SD = 1.8; *t*(17.98) < 1]. However, the controls produced few errors (mean number of errors left after artifact rejection in the Naming task = 15, SD = 10, with one participant having less than five error trials left for EEG signal averaging; mean number of errors in the Simon task = 10, SD = 8, with two participants having less than five error trials left for EEG signal averaging). No clear Ne was observed in the controls due to poor signal-to-noise ratios and their EEG data for errors were not analyzed further, although we report their EEG data in correct trials. We note patients had on average 28 errors (SD = 16) left after artifact rejection in the Naming task and 23 (SD = 23) in the Simon task.

Lesion etiology was stroke in all patients and they were recorded at least 6 months post-stroke. Their lesions were centered in the left IFG and MFG and right MFG. Importantly, their medial frontal cortex (MFC) and posterior STG were spared although one left and one right PFC patients had larger lesions including the superior anterior quadrant of the left and right temporal lobe, respectively. None of the patients or controls had any other neurological or psychiatric diagnoses.

All participants were native English speakers, had normal or corrected-to-normal vision and all but one patient and matched control were right-handed. Importantly, no language impairment was diagnosed by neurological assessment in the left-handed patient with right PFC lesion and there was no history of left-handers in the family, suggesting this patient was probably left-hemisphere dominant for language. This patient did not show impairment in either task.

The study was performed in agreement with the Declaration of Helsinki. All subjects gave informed consent approved by the University of California, Berkeley Committee for Protection of Human Subjects and the Department of Veterans Affairs Northern California Health Care System Human Research Protection Program or the Department of Veterans Affairs New Mexico Health Care System Human Research Protection Program. Participants received remuneration for their participation.

### MATERIAL AND DESIGN

The stimuli were line drawings of common objects or animals selected from a published collection (Snodgrass and Vanderwart, 1980) or constructed by us for this experiment. Their name agreement was tested on a set of 10 controls whose data were not included in the experiment but whose mean age was not significantly different from the set of patients tested here [*t*(18.06) = 1.42, *p* = 0.17; mean name agreement: 91.25%, SD = 8%]. They were all 525 × 250 pixels high and were presented in free viewing within a visual angle of 7°. A total of 252 pictures were used: 216 were the experimental items and 36 were used as practice trials. For purposes unrelated to the present study, the pictures were issued from six semantic categories (e.g., animals), each member (e.g., cat) was represented by six different items (e.g., six different cats), and they were presented within semantically related vs. unrelated blocks in similar fashion as in Damian et al. (2001). In addition, because participants also performed a Simon task (Craft and Simon, 1970) the pictures were colored in green or purple and could be presented on the left or the right of the fixation point.

### PROCEDURE

Participants were tested in a sound-attenuated dimly lit environment. They were seated comfortably 148 cm from a computer screen on which the stimuli were displayed. The experiment was controlled by the Eprime 2.0 Professional software (Psychology Software Tools, Inc., Pittsburgh, PA, USA), which allows on-line recording of the participants’ verbal responses.

A trial consisted of the following events: (1) a fixation point (“plus” sign presented at the center of the screen) for 500 ms; (2) a picture for 2000 ms (3) a blank screen for 2000 ms. The following trial started automatically. Participants performed two tasks in separate blocks: a picture naming task and a verbal Simon task. In the Naming task, participants were asked to name the picture by saying the name of the picture preceded by the possessive determiner “my” (e.g., “my cat”). In the Simon task, participants were asked to say “my right” or “my left” depending on the color of the picture while ignoring the side to which the picture was presented. Thus interference is greater for a picture presented on the left of the fixation cross when the response to be given is “my right” and vise-versa. The stimulus-response association rule (i.e., saying “my right” for a green picture and “my left” for a purple picture or vise-versa) and the order in which the tasks were performed were counterbalanced across participants.

The possessive determiner was added to reduce variability in vocal onsets and because we also recorded EMG activity of three facial articulators. Since the nature of the first phoneme influences the EMG activity pattern (Riès et al., 2012), we chose to have all utterances start with the same phoneme. EMG was recorded in an attempt to observe clearer response-locked components. However, EMG could not be recorded or was too noisy in three of the patients because of facial hair or difficulty in relaxing facial muscles. Because of the small number of errors patients made overall, discarding more trials because of EMG recording problems would have left too few trials for further analysis. We therefore do not report EMG analyses in this study.

Vocal-onsets were used as the response-onset measure. Each task was split into 4 blocks of 108 trials each, with two pauses equally spaced within each block. Participants performed all four blocks of one task before the four blocks of the other task. Altogether, the participant saw the same item four times corresponding to the four possible color/side configurations: green on the left, green on the right, purple on the right, and purple on the left. The type of configuration seen per task and per type of block was counterbalanced across participants.

The participants were asked to give their response verbally as fast and as accurately as possible. Participants were informed that no correction was possible in the case of errors (Vidal et al., 2000; Riès et al., 2011). Participants were also asked to remain as relaxed as possible and to avoid making movements that could generate artifacts on the EEG (e.g., eye blinks, frowning) during the trials. The experiment consisted in two parts per task. First participants were familiarized with the name of the pictures to be seen in the experiment and with the Simon task. Instructions were given and the experimenter made verbal corrections when an incorrect or unexpected response was produced. We wanted to avoid visual habituation to the experimental stimuli and thus used a set of 36 pictures consisting of a seventh exemplar of each member of each category used in the experiment. Importantly, these practice items had the same names as the experimental stimuli. The pictures were presented one by one in a random order and were displayed in the same manner as the experimental stimuli. Second, the experimental instructions were delivered and the experiment started. The experimental session lasted for an hour to an hour and a half depending on the length of the breaks.

### ELECTROPHYSIOLOGICAL RECORDINGS

The EEG was recorded from 64 Ag/AgCl pre-amplified electrodes (BIOSEMI, Amsterdam, Netherlands; 10–20 system positions). The sampling rate was 1024 Hz (filters: DC to 208 Hz, 3 db/octave). The vertical electro-oculogram (EOG) was recorded by means of two surface electrodes just above and below the left eye, respectively. The horizontal EOG was recorded with two electrodes positioned over the two outer canthi. The passive reference was placed over the left mastoid.

### DATA PRE-PROCESSING

#### Behavioral data pre-processing

The accuracy of the responses and the verbal reaction times were measured offline using the software CheckVocal (Protopapas, 2005). Trials were excluded from the analysis of the correct responses if the participant did not respond, or produced any kind of verbal error: partial or complete production of incorrect words, omission of the pronoun “my,” verbal disfluencies (stuttering, utterance repairs, etc.), and hesitations (e.g., if the experimenter perceived the production of the possessive pronoun to be abnormally lengthened or separated from the production of the noun by a pause). Verbal errors but not no-responses were included in the analysis of errors. All the errors were coded in a single category. Incorrect trials could also be made of two utterances if the participant attempted to correct him/herself despite instructions. Importantly, the marker indicating the onset of the error was always placed at the beginning of the sound waveform of the first recorded utterance (as reported in Riès et al., 2011).

#### EEG Data pre-processing

After acquisition, the EEG data were filtered (high pass = 0.16 Hz) and resampled at 256 Hz. Vertical eye movements were corrected based on an independent component analysis as implemented in EEGLAB (Delorme and Makeig, 2004). Speaking induces large facial EMG activities that contaminate the EEG signal. To reduce the EMG artifacts induced by articulation, we used a Blind Source Separation algorithm based on Canonical Correlation Analysis (BSS-CCA, De Clercq et al., 2006) that separates sources based on their autocorrelation. The suitability of BSS-CCA for removing articulatory EMG bursts from EEG signal is described in detail in De Vos et al. (2010) and was used successfully to study monitoring-related components in Riès et al. (2011). In the current study, we used the BSS-CCA method similarly as reported in Riès et al. (2011) except the length of the non-overlapping consecutive windows was 2 s, corresponding to the duration of a trial.

Following the BSS-CCA procedure, all other artifacts were rejected on the basis of a trial-by-trial visual inspection of monopolar recordings. The retained monopolar recordings were averaged and time-locked to vocal-onset. Laplacian transformation (i.e., current source density, C.S.D., estimation), as implemented in BrainAnalyser^TM^ (Brain Products, Munich), was applied to each participant’s averages and on the grand average as in Riès et al. (2011); (degree of spline: 3, Legendre polynomial: 15° maximum). We assumed a radius of 10 cm for the sphere representing the head. The resulting unit was μV/cm^2^. A 30-Hz low-pass and 1-Hz high-pass filters were applied off-line on the EEG data.

### ANALYSIS

The analysis included the factor “accuracy” (correct or error) and “participants” as a random effect. The behavioral data were analyzed using Student’s *t*-tests or ANOVAs for comparison of more than two means. When unpaired *t*-tests were performed, a Welch correction for non-homogeneity of variance was applied.

The main analysis for EEG data was performed for the signal recorded at electrode FCz, where Ne-like waves are typically observed (Vidal et al., 2000), and at T7, TP7, TP8, and T8, over the left and right temporal cortices as these regions have also been associated with speech monitoring. Statistical analyses were performed on the slopes of the activities on 100–50 ms time-windows preceding the peaks of interest (depending on the size of the activity on the grand averages), peak-to-peak amplitudes, and peak latencies of Laplacian-transformed data. The statistical reliability of the activities was assessed by comparing the slopes of the waveforms (measured with linear regression fit) to zero across subjects. The time-window used for these slope analyses were determined based on the shape of the waveform on the grand averages. Peak latencies were measured on smoothed data (length of the smoothing window: 40 ms) to minimize the impact of background noise. Peak-to-peak amplitudes were measured by first measuring the surfaces below the waveforms on 40 ms time windows around the peak latency per subject, and then by subtracting these surface values to one another as described in Riès et al. (2011), 2013. All these measures were compared using non-parametric exact Wilcoxon signed-rank tests (i.e., Wilcoxon *t*-tests) or Kruskal–Wallis rank sum test for comparison of more than two means because the measures were based on few error trials and the normality of the data could not be assumed. Following Siegel (1956), we reported Wilcoxon *t*-values, corresponding to the sum of the absolute values of the ranks of the least represented sign, and the associated *p*-values, and Kruskal–Wallis *H*-values and corresponding *p*-values. All statistical analysis were performed using R 2.15.2 (R Core Team, 2012).

## RESULTS

### BEHAVIORAL DATA

Overall, 3.26% (SD = 3.26%) of the trials were removed from further analysis due to no responses. This rejection rate was higher in the Naming task (4.01%, SD = 3.16%) than in the Simon task [2.50%, SD = 4.16%, *F*(1, 22) = 4.53, *p* = 0.045] but was not significantly higher for patients than for controls [*F*(1, 22) = 2.29, *p* = 0.145]. There was no main effect of lesion side in patients [*F*(1, 10) < 1] and only a marginal interaction between lesion side and task [*F*(1, 10) = 4.21, *p* = 0.067] in that left PFC patients had less trials rejected in the Simon task (1.08%, SD = 1.19%) than in the Naming task (5.59%, SD = 3.78%) but the right PFC patients did not (Naming: 5.16%, SD = 4.11%; Simon: 5.09%, SD = 7.32%).

The average error rate (i.e., percentage of errors) was higher for patients (Naming: 7.56%, SD = 3.78%; Simon: 6.00%, SD = 5.22%) than for controls [Naming: 5.27%, SD = 3.57%; Simon: 3.03%, SD = 2.50 %; *F*(1, 22) = 4.49, *p* = 0.046] and tended to be higher in the Naming than in the Simon task [*F*(1, 22) = 3.70, *p* = 0.067]. There was no group by task interaction [*F*(1, 22) < 1]. There was no effect of lesion side in patients *t*[*F*(1, 10) < 1], nor any interaction of lesion side with task [*F*(1, 10) = 2.70, *p* = 0.131]. Overall, participants self-corrected their responses despite instructions in only 0.8% of trials.

The average RT for correct trials was longer in the Naming than in the Simon task for both patients and controls [*F*(1, 22) = 10.43, *p* = 0.004]. For patients, the average RT was 816 ms (SD = 89 ms) in the Naming task, and 782 ms (SD = 109 ms) in the Simon task. For controls, the average RT was 798 ms (SD = 105 ms) in the Naming task and 740 ms (SD = 104 ms) in the Simon task. Patients were not significantly slower than controls [*F*(1, 22) < 1] and there was no effect of lesion side on average RTs [*F*(1, 10) < 1] nor any interaction between lesion side and task [*F*(1, 10) < 1]. Overall, two controls (two females) and two patients (one left PFC, one right PFC, one male, one female) made less than five errors in one or both tasks. Their data were not included in the following analyses of RTs in errors and comparison between RTs in errors and correct trials leaving 10 controls, 5 right and 5 left PFC patients for these analysis (these groups still did not differ in terms of age or education: *t*s < 1). The average RT for errors for patients in the Naming task was 918 ms (SD = 133 ms) and it was 872 ms (SD = 105 ms) in the Simon task. The average RT for errors for controls in the Naming task was 867 ms (SD = 192 ms) and it was 825 ms (SD = 144 ms) in the Simon task. There was no difference between patients and controls on error RTs [*F*(1, 18) < 1] nor any main effect of task [*F*(1, 18) = 1.41, *p* = 0.250] or interaction between these factors [*F*(1, 18) < 1]. There was no effect of lesion side [*F*(1, 8) < 1] on error RTs. RTs were significantly longer in errors than in correct trials in patients and controls for both tasks [*F*(1, 18) = 32.35, *p* < 0.0001], there was no interaction between lesion side and accuracy [*F*(1, 8) < 1].

### EEG DATA

As noted, control participants had diminished signal-to-noise ratio in the EEG averages for errors and we report the EEG data of the patients for incorrect and correct trials and of the controls only for correct trials. One left frontal patient had less than 40% of trials left after artifact rejection and was removed from the following analysis. The remaining participants (*N* = 11: 5 left, 6 right PFC, still matched in age and gender to the controls: *t*s < 1) had on average 84% (SD = 11%) of trials left after artifact rejection. This group of 11 patients was used for the analysis of correct trials in the Naming and Simon tasks and errors in the Naming task. A group of eight patients was used for the analyses of errors and the comparison of errors with correct trials in the Simon task (excluding two left and one right PFC patients who had less than five epochs left for averaging after artifact rejection in errors, again not differing from the control group in age or education: *t*s < 1).

#### EEG Data in patients

**FIGURE 2:**
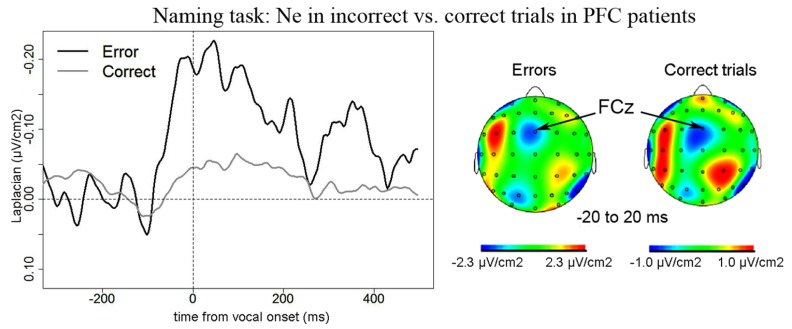
**EEG activity in PFC patients in the Naming task after surface Laplacian transformation, recorded at FCz for errors (black line) and correct trials (gray line).** Zero of time represents vocal onset. The cartographies were made on a 40-ms time-window centered on vocal onset (from -20 to 20 ms after vocal onset). A 100 ms-long baseline was taken between 200 and 100 ms before vocal onset. The scale used for the topography for correct trials was larger than the one used for incorrect trials as the amplitude of the Ne was smaller in correct trials than in errors.

**FIGURE 3:**
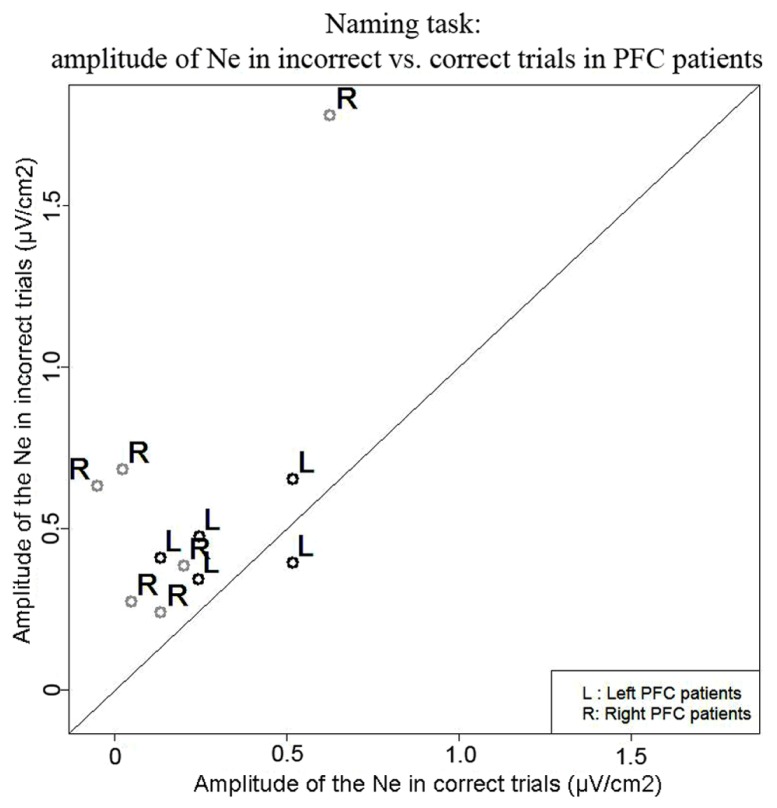
**Comparison of the amplitude of the Ne in correct (*x* axis) vs. error (*y* axis) trials in the Naming task.** Each point represents a patient, points for left PFC patients are black and indicated by the letter “L” and points for right PFC patients are gray and are indicated by the letter “R.”

***Naming task.*** We observed a Ne for error and correct trials. The negativities peaked slightly after vocal onset and were associated with a fronto-central local topography (**Figure 2**). The slopes of the waveforms were below zero on the 100-ms time-window preceding vocal onset for both types of trials [error trials: *t*(11) = 7, *p* = 0.009; correct trials: *t*(11) = 11, *p* = 0.027, one-sided Wilcoxon *t*-tests were used as the direction of the difference was expected based on previous reports, e.g., Vidal et al., 2000; Riès et al., 2011]. The Ne peaked in average 33 ms (SD = 61 ms) after vocal onset for error trials and 67 ms (SD = 70ms) after vocal onset for correct trials. There was no significant difference in latency between the Ne in errors and in correct trials [*t*(11) = 17, *p* = 0.175]. The latency of the preceding positivity was not different between errors and correct trials [*t*(11) = 17, *p* = 0.175; errors: mean = -166ms, SD = 80 ms; correct trials: mean = -128 ms, SD = 64 ms]. Critically, the peak-to-peak amplitude between the Ne and the preceding positivity was larger for incorrect than correct trials [*t*(9) = 63, *p* = 0.005; Mean for errors = 0.57 μV/cm^2^, SD = 0.43 μV/cm^2^; Mean for correct trials = 0.24 μV/cm^2^, SD = 0.22 μV/cm^2^]. As can be seen on **Figure 3** (showing the individual data), this was true for all but one patient. We did not have a sufficient number of left vs. right patients to test for the effect of lesion side on the amplitude of the Ne. Interestingly, the only patient who did not have a larger Ne in errors vs. correct trials was the one with a large lesion including part of the left temporal lobe, roughly the superior anterior quadrant.

**FIGURE 4:**
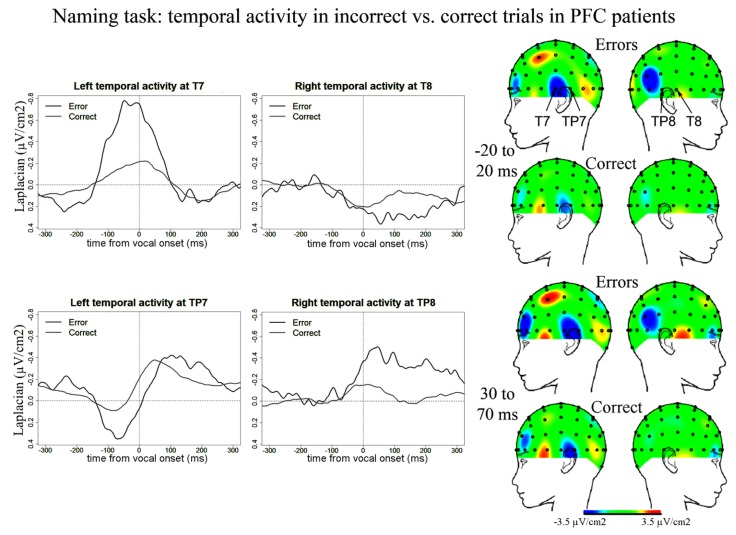
**EEG activity in PFC patients after surface Laplacian transformation in the Naming task, recorded at left (T7, TP7) and right (T8, TP8) temporal electrodes for error (black line) and correct trials (gray line).** Zero time represents vocal onset. The cartographies were made on 40 ms time-windows centered on vocal-onset and 50 ms after vocal-onset. The baseline was taken between 200 and 100 ms before vocal onset. The same scale was used for all topographies.

We also report activity at left and right temporal recording sites (electrodes T7, TP7, TP8) peaking around and after vocal-onset which was larger for error than correct trials (**Figure 4**). Negative components in errors peaked on average 55 ms (SD = 88 ms) after vocal-onset at T7, 71 ms (SD = 68 ms) at TP8 and 125 ms (SD = 67 ms) at TP7. In correct trials, negative components peaked on average 17 ms (SD = 41 ms) after vocal onset at T7, 18 ms (SD = 47 ms) at TP8 and 63 ms (SD = 35 ms) at TP7. There was an effect of accuracy on the latencies at which these components reached their maximum [*t*(11) = 109.5, *p* = 0.002], latencies were longer in errors than in correct trials. This effect was due to a significant difference in latencies at TP7 only [*t*(11) = 55, Bonferroni corrected *p*-value = 0.018]. The slopes of the waveforms were significantly below zero for error trials [*t*(11) = 99, *p* < 0.001] with a trend for correct trials [*t*(11) = 191, *p* = 0.056, measures made on 100 ms time-windows within the rising of the negativity, from -100 ms until vocal-onset for T7 and from -50 until 50 ms for TP7 and TP8, all three recording sites were pooled in these analyses, two-sided Wilcoxon *t*-tests were used]. The peak-to-peak ]amplitudes were greater for error than correct trials [*t*(11) = 149, *p* = 0.018]. There was no significant activity at T8 for error trials [slopes not different from zero: *t*(11) = 44, *p* = 0.365] or correct trials [*t*(11) = 53, *p* = 0.083] despite a positive slope observed on the grand averages. As can be seen on the topographies, the right-sided temporal activity was reduced compared to the left side in both correct and incorrect trials.

**FIGURE 5:**
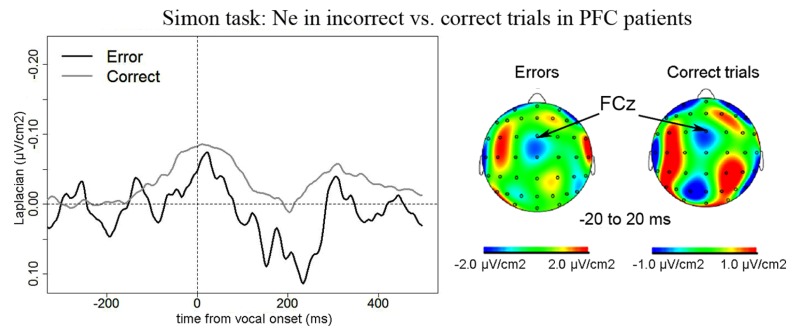
**EEG activity in PFC patients in the Simon task after surface Laplacian transformation, recorded at FCz for errors (black line) and correct trials (gray line).** Zero of time represents vocal onset. Similarly as for the Naming task, the cartographies were made on a 40-ms time-window centered on vocal onset (from -20 to 20 ms after vocal onset) and a 100 ms-long baseline was taken between 200 and 100 ms before vocal onset.

**FIGURE 6:**
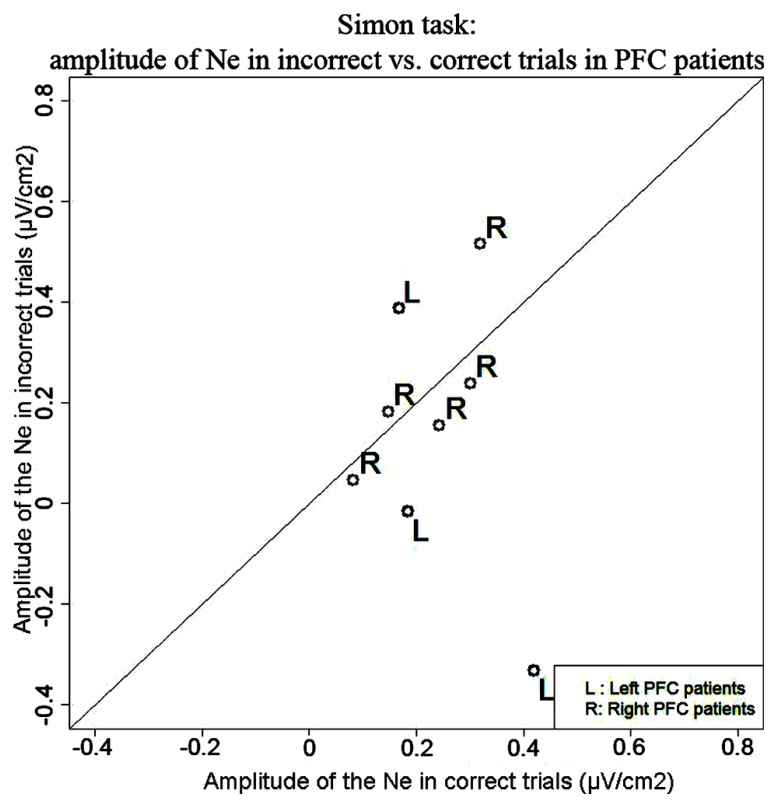
**Comparison of the amplitude of the Ne in correct (*x* axis) vs. error (*y* axis) trials in the Simon task.** Each point represents a patient, points for left PFC patients are black and indicated by the letter “L” and points for right PFC patients are gray and are indicated by the letter “R.”

***Simon task.*** We observed a Ne for correct trials but not reliably for incorrect trials. On correct trials, the Ne peaked on average 37 ms after vocal onset (SD = 60 ms) and was associated with a fronto-central local topography (**Figure 5**). The slopes of the waveforms were below zero on the 100-ms time-window preceding vocal onset for correct trials [*t*(11) = 6, *p* = 0.007] but not for error trials [*t*(11) = 13, *p* = 0.273]. We note the number of error trials left after artifact rejection was not lower in the Simon task (average *n* = 30, SD = 23, for the eight patients having more than five errors left after artifact rejection) than for the Naming task [average *n* = 28, SD = 16; *t*(12.03) < 1, *p* = 0.82, an unpaired Student *t*-test was used given the different number of patients in each group]. Moreover, when the data of the three patients who had less than five error trials left after artifact rejection in the Simon task were also removed from the slope analysis of the error trials in the Naming task, there was still a reliable Ne apparent [*t*(8) = 5, *p* = 0.039]. Thus, the difference in patterns we observe in errors is not likely due to a lower signal-to-noise ratio in the Simon task. To be able to compare errors to correct trials in the Simon task, we used the same latency values in errors as in correct trials to measure the peak-to-peak amplitudes in errors. There was no significant difference in the peak-to-peak amplitudes thus measured between errors and correct trials [*t*(8) = 22, *p* = 0.641, see **Figure 6**]. The interaction between task and accuracy on these peak-to-peak amplitudes was not significant (*H* = 1.841, *P* = 0.173).

**FIGURE 7:**
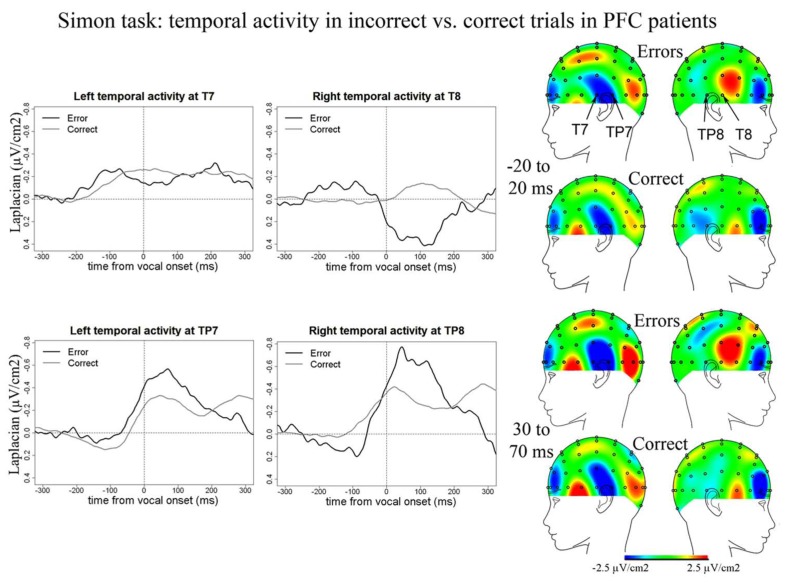
**EEG activity in PFC patients after surface Laplacian transformation in the Simon task, recorded at left (T7, TP7) and right (T8, TP8) temporal electrodes for error (black line) and correct trials (gray line).** Zero time represents vocal onset. The cartographies were made on 40 ms time-windows centered on vocal-onset and 50 ms after vocal-onset. The baseline was taken between 200 and 100 ms before vocal onset. The same scale was used for all topographies.

We also observed temporal activity peaking around vocal onset in the Simon task in PFC patients (**Figure 7**). In errors, negative peaks of activity were reached 187 ms (SD = 64 ms) post-vocal onset at T7, 41 ms (SD = 51 ms) at TP8, and 18 ms (SD = 49 ms) at TP7. In correct trials, negative peaks of activity were reached at 172 ms (SD = 114 ms) post-vocal onset at T7, 26 ms (SD = 63 ms) at TP8, and 30 ms (SD = 72 ms) post-vocal onset at TP7. There was no effect of accuracy on these latencies [*t*(8) = 151.5, *p* = 0.977]. We note two peaks of activity are observed on grand averages at T7 in errors. We considered the highest peak of activity for the latency measures and peak-to-peak measures. The slopes of the waveforms were smaller than zero in errors [*t*(8) = 36, *p* < 0.001] and correct trials at T7, TP8, and TP7 [*t*(11) = 162, *p* = 0.034; slopes were measured on 100 ms long time-windows comprised within the rise of the negativities on the grand averages]. Critically and in contrast with the Naming task, there was no difference in amplitude between errors and correct trials for these temporal activities in the Simon task [*t*(8) = 129, *p* = 0.565, none of the pairwise comparisons were significant: all bonferroni-corrected *p*s > 0.16]. We note we also observed a positivity at a right temporal site (T8) peaking on average 99 ms after vocal onset (SD = 66 ms) for error trials [slopes were different from zero: *t*(8) = 33, *p* = 0.039] but not for correct trials where the slope of the waveform was not significantly different from zero [*t*(11) = 45, *p* = 0.320].

#### EEG Data in controls

**FIGURE 8:**
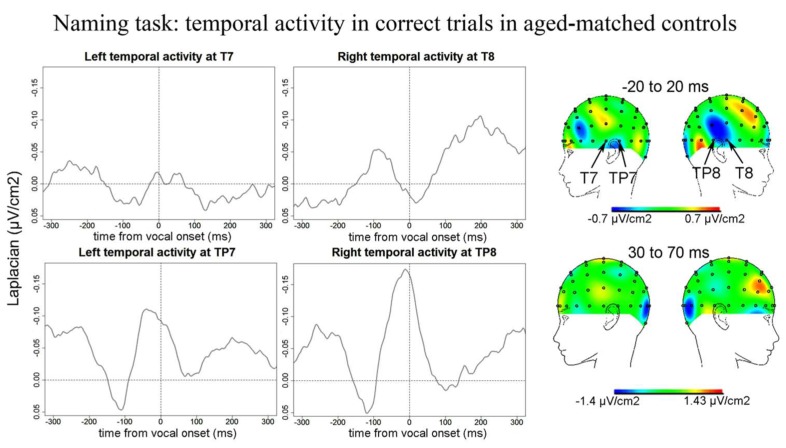
**EEG activity for control group after surface Laplacian transformation, recorded at left (T7, TP7) and right (T8, TP8) temporal electrodes for correct trials in the Naming task.** Zero time represents vocal onset. The cartographies were made on a 40-ms time-window centered around vocal-onset and 50 ms after vocal-onset. The baseline was taken between 200 and 100 ms before vocal onset.

***Naming task.*** No Ne for correct trials was reliably observed at fronto-central sites in the Naming task. We report activity at left and right temporal recording sites for correct trials in controls peaking around vocal onset (**Figure 8**): on average 5 ms after vocal onset at T7 (SD = 35 ms), 20 ms before vocal onset at TP8 (SD = 32 ms), and 34 ms before vocal onset at TP7 (SD = 36 ms). The slopes of the waveforms were different from zero [*t*(12) = 206, *p* = 0.023; 100 ms time-windows spanning from -100 ms to vocal onset were used for TP7 and TP8 and a 50 ms time-window spanning from -50 to vocal-onset was used for T7 given the shape of the activity on the grand average, T8 was excluded from the analysis as no activity peaked around vocal onset at that recording site]. Although the negativity peaking around vocal onset at TP8 appeared bigger than the negativity peaking at TP7, there was no statistical difference between the peak-to-peak amplitudes measured [*t*(12) = 38, *p* = 0.970]. We did note the presence of a component at CP6, just superior to TP8 that seemed absent at the contralateral site (CP5). We compared the peak-to-peak amplitudes measured for correct trials in patients to those measured in controls. Peak-to-peak amplitudes were marginally smaller in controls than in patients (*t* = 438, *p* = 0.061). We also note that the activity at TP7 did not persist after vocal onset as in patients, the cartographies at 50 ms after vocal onset did not show any activity over left or right temporal cortices.

**FIGURE 9:**
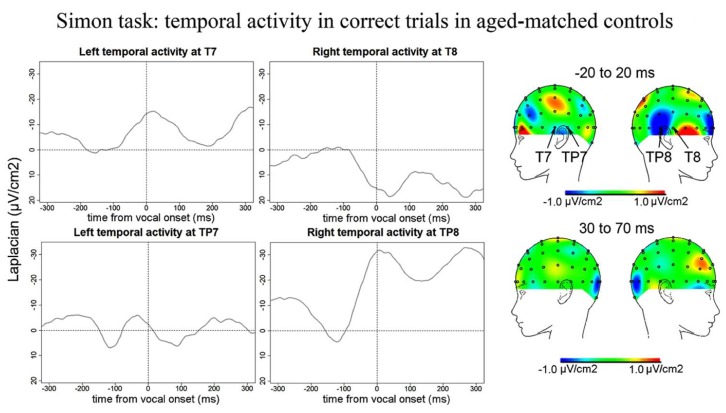
**EEG activity for control group after surface Laplacian transformation, recorded at left (T7, TP7) and right (T8, TP8) temporal electrodes for correct trials in the Simon task.** Zero time represents vocal onset. The cartographies were made on a 40-ms time-window centered around vocal-onset and 50 ms after vocal-onset. The baseline was taken between 200 and 100 ms before vocal onset.

***Simon task.*** Similarly to the Naming task, no reliable Ne was observed at fronto-central sites for the Simon task. We report activity at left and right temporal recording sites peaking around vocal-onset (**Figure 9**): at 51 ms (SD = 66 ms) after vocal-onset on average at T7, at 38 ms (SD = 69 ms) after vocal-onset at TP8, and at 2 ms (SD = 89 ms) before vocal onset at TP7. The slopes of the waveforms were inferior to zero [*t*(12) = 98, *p* < 0.001; 100 ms time-windows spanning from -100 ms to vocal onset were used for T7 and TP8 and a 50 ms time-window spanning from -100 to -50 ms before vocal-onset was used for TP7 given the shape of the activity on the grand average]. On the grand averages, a positivity can be seen at T8 but the slope of the waveform was not significantly different from zero on the 100 ms preceding vocal-onset [*t*(12) = 37, *p* = 0.910]. Although the right temporal activity at TP8 seemed larger than the left temporal activity at T7 and TP7, there was no effect of recording site on the peak-to-peak amplitude measured (*H* = 2.72, *P* = 0.25, none of the two-by-two comparisons were significant either). Peak-to-peak amplitudes were not smaller in controls than in patients (*t* = 522, *p* = 0.393).

## DISCUSSION

In agreement with our hypothesis, we observed an amplitude difference between the Ne for error and correct trials in PFC patients in overt picture naming but not in the verbal Simon task. This suggests the domain-general monitoring process supported by MFC and reflected by the Ne is not as dependent on the LPFC when no arbitrary rule has to be maintained in working memory (Gehring and Knight, 2000; Ullsperger et al., 2002). The characteristics of the Ne for error in Naming and correct trials in both tasks in PFC patients corresponded to those described in young controls (Riès et al., 2011) and differed in amplitude for error and correct trials in Naming as in young controls but not in the Simon task. Peak latencies of the negativity, did not differ for error and correct trials, and the fronto-central scalp topographies associated with the Ne were also similar for both trial types. There were minor differences noted which we discuss in more detail below. In addition, we observed activity recorded at electrodes over temporal cortices starting before and peaking around and after vocal-onset. This activity was larger for error than correct trials in PFC patients only in Naming, suggesting it is also associated with on-line speech monitoring when access to lexical representations is necessary.

### ROLE OF THE LATERAL PFC IN SPEECH MONITORING

Though the LPFC, and especially the inferior part of the left PFC (BA44/45), has been associated with a range of language deficits, our results suggest that its involvement in speech monitoring as assessed by simple picture naming is not as critical as in other actions in which an arbitrary rule has to be maintained in working memory. Patients made more errors than controls overall, suggesting both tasks were harder for patients than for controls. However for patients, the Ne was larger in errors than in correct trials in Naming but not in the Simon task. This suggests that despite having more difficulty overall, the patients’ monitoring system was relatively preserved in the Naming task but not in the Simon task. In our study, the slope analysis failed to reveal a significant component in errors in the Simon task. However, the absence of an amplitude difference replicates the pattern of results reported in Gehring and Knight (2000) and Ullsperger et al. (2002) and Ullsperger and von Cramon (2006). These studies have reported abnormal Ne patterns in patients with lesions to the LPFC using non-linguistic decision-making tasks: both studies report no amplitude difference between the Ne reported in errors vs. correct trials. We note there were some relative amplitude differences in errors vs. correct trials between the two studies, as Gehring and Knight (2000) reported an abnormally large Ne in correct trials in PFC patients compared to controls whereas Ullsperger et al. (2002) reported a reduced Ne in errors in PFC patients compared to controls. Such a difference may be explained by patient variability (the patients tested in Gehring and Knight’s study were in average almost 20 years older than those tested in Ullsperger et al.’s) but also by the fact that slightly different tasks were used (see below). We propose that the different pattern of results obtained in non-linguistic tasks compared to our Naming task may be linked to the different nature of the stimulus-response associations.

In our Simon task as in Gehring and Knight (2000) and in Ullsperger et al. (2002) and Ullsperger and von Cramon (2006) studies, the tasks used involved arbitrary stimulus-response associations that had to be maintained in PFC-dependent working memory. In the Simon task, the response to be given depends on an arbitrary association between the color of the stimulus and the response. The two other studies mentioned used modified versions of the Eriksen Flanker task (Eriksen and Eriksen, 1974) in which participants are instructed to press left or right buttons depending on the central letter (H or S) of a string of three or five letters. Responding is more difficult if the letters flanking the central letter are associated with the incorrect response (e.g., HSH) than if all letters displayed are associated with the correct response (e.g., HHH; Ullsperger et al., 2002; Ullsperger and von Cramon, 2006), used the “arrow”-version of the Flanker task in which letters are replaced by arrows pointing right or leftward). In order to make the task even more difficult and elicit more errors, Gehring and Knight (2000) used an additional task switching component and Ullsperger et al. (2002) and Ullsperger and von Cramon (2006) used a speeded version of the task.

Simple picture naming does not require the participants to hold an abstract rule into working memory as the link between the stimulus and the response to be made (i.e., the name of the picture) is subserved by lexical access to long term memory. The importance of the LPFC in holding an abstract rule or goal in working memory has been reported by numerous neuropsychological and neuroimaging studies (for reviews see Miller and Cohen, 2001; Ridderinkhof et al., 2004a, 2010). In addition, the relative roles of the MFC (especially the ACC) and the LPFC have been empirically contrasted (e.g., MacDonald et al., 2000). These authors used a Stroop paradigm containing a task-switching component: whereas the ACC was associated with conflict monitoring within trials of the Stroop task, the DLPFC was associated with task switching demands. We suggest that the different pattern we report between the Naming and the Simon tasks is linked to this fundamental difference in the nature of the task performed. If true, increasing the working memory load in a linguistic task may lead to similar results as the ones observed in non-linguistic tasks.

An alternate explanation for the task-dependent pattern observed is related to differences in the nature of response competition in both tasks. The naming task we used involved a semantic context manipulation known to elicit a semantic interference effect. Participants are slower to name pictures when pictures are presented in a semantically related vs. unrelated context. This effect is often interpreted as reflecting competition between semantically related alternatives at the level of lexical selection (e.g., Damian et al., 2001; Belke et al., 2005; although see Oppenheim et al., 2009). In the Simon task, an irrelevant aspect of the stimulus, namely its position, competes with the representation of the response given on half of the trials. The type of interference differs in that the semantic interference effect builds up from trial to trial whereas in the Simon task, incompatibility between the response to be given and the stimulus position occurs within a trial. Ganushchak and Schiller (2008a) have reported an effect of semantic context on the Ne but the evidence is not as clear for the Simon task (Masaki et al., 2007). Though both interference effects have generally been attributed to stages of processing taking place upstream from response monitoring (see e.g., Damian et al., 2001, for the semantic interference effect; and Burle et al., 2002, 2008 for Simon-type interference) and earlier components have been shown to be affected by these manipulations (e.g., Maess et al., 2002; van der Lubbe and Verleger, 2002; Aristei et al., 2011), interference linked to semantic context could also secondarily affect speech monitoring (as suggested by Ganushchak and Schiller, 2008a). However, this would lead to an opposite pattern as the one we observe. Indeed, here the abnormal pattern was in the Simon task, not in the Naming task. Thus, we believe our pattern of results is best explained by differences in working memory requirements between the two tasks although further investigation is necessary to fully address this issue.

We also note that recruitment of LPFC regions has been shown to be dependent on task difficulty (Mostofsky et al., 2003). However, the different pattern of results we report in the Naming task compared to the Simon task is unlikely due to this factor as the error rate was not higher in Naming than in the Simon task and in fact there was a trend for a higher error rate in the Naming than in the Simon task.

### TEMPORAL ACTIVITY

We observed temporal activity starting before and peaking around vocal onset in correct trials in both patients and controls and also in errors in patients in both tasks. Critically, these temporal components were larger in errors than in correct trials in patients in Naming but not in the Simon task, suggesting they were associated with speech monitoring when linguistic representations are accessed. Indeed, an important difference between the Naming and the Simon tasks is that lexical access is much reduced in the Simon task compared to the Naming task as only two words (“left” and “right”) have to be articulated in the Simon task.

The observation that temporal activity starts before vocal onset in both patients and controls supports a role in inner speech monitoring, before overt auditory feedback can be perceived as suggested in Riès et al. (2011). The left temporal activity reported in Riès et al. (2011) also started before vocal onset although it peaked later, around 200 ms post vocal onset. In our study, the components recorded at T7 peaked just around vocal-onset. We also report components at electrodes just posterior to T7 and T8 (TP7 and TP8). In patients in the Naming task, the negativity at TP8 reached its maximum around 30 ms post vocal onset and then decreased rapidly but at TP7, the negativity reached its maximum at around 70 ms post vocal onset and then seemed to decrease more slowly than at the contralateral site. The left but not the right activity was still visible on the scalp cartography 50 ms after the response, suggesting left temporal activity is also engaged in outer speech monitoring.

Bilateral temporal activity was also visible in correct trials in aged-matched controls although it was marginally smaller in the Naming task and had a slightly different time-course. Indeed, there was no more visible activity on the scalp cartographies over the left or right temporal cortices 50 ms after response onset. This leads to two possible interpretations. First, LPFC patients relied on the mechanism underlying this brain activity, linked to both inner and outer speech monitoring, more than aged-matched controls. This seems plausible as patients had more difficulty in performing the task than controls. An alternate interpretation may be that this mechanism was affected in the patients. Indeed, PFC patients may have difficulty in resolving competition at the level of lexical selection. As mentioned below, different regions of the left temporal cortex have been associated with lexical selection (e.g., Trebuchon-Da Fonseca et al., 2009; Baldo et al., 2013). It has been suggested that such competition is harder to overcome in PFC patients and especially in left PFC patients (e.g., Thompson-Schill et al., 1997, 1998). As previously discussed we failed to report differences in performance between left and right PFC patients and the error rate was too small to test for such differences in the EEG data. Thus, based on the present study, we cannot determine which of these two proposed interpretations can explain the longer lasting temporal activity in Naming in patients vs. controls.

The reason why the pattern of activity we report in older patients and controls is somewhat different than what was reported in young controls is also uncertain. This might again be due to the use of the possessive determiner in the present study and not in Riès et al. (2011). Another possibility could be linked to aging. Aging has been associated with different Ne patterns in non-linguistic tasks (e.g., Gehring and Knight, 2000) such that the difference between errors and correct trials was smaller than in young controls. Other components of the network associated with speech monitoring could also be affected. Moreover, older controls have been shown to use different error repair strategies than young controls in speech production (McNamara et al., 1992), suggesting that their monitoring system did not lead to similar behaviors.

Although valuable information is gained from the fine temporal resolution available with EEG, its limited spatial resolution does not enable certainty as to which brain region(s) is/are generating the activity we report. The focal lateral scalp topography supports a generator in temporal cortices but we cannot be more specific on the basis of this study. The posterior STG has been associated with speech monitoring, and especially external speech monitoring based on fMRI studies manipulating auditory feedback (e.g., McGuire et al., 1996; Hashimoto and Sakai, 2003; Fu et al., 2006; Tourville et al., 2009). Because pSTG is also one of the main regions associated with speech perception, activation in this region in situations in which the monitoring of auditory feedback is made more difficult has generally been interpreted as supporting the perceptual loop account of speech monitoring (see Indefrey and Levelt, 2004; Indefrey, 2011). Thus an association is often made between temporal lobe activation, external speech monitoring and speech comprehension. Our data suggest that temporal lobe activations are also associated with inner speech monitoring. However, we do not believe that our results can validate or invalidate the perceptual-loop theory according to which both inner and outer speech monitoring rely on the speech comprehension system (Levelt, 1983). Indeed, other parts of the temporal lobes, and especially the left temporal lobe, have been associated with language production processes. Notably, the middle temporal gyrus (MTG) and left posterior and basal temporal regions (Brodmann’s area 20-37-39) have been linked to lexical access in overt speech production (e.g., Trebuchon-Da Fonseca et al., 2009; Baldo et al., 2013). It is possible that what we are observing is increased activation within these production-related areas in errors vs. correct trials and in LPFC patients vs. aged-matched controls. This would be in agreement with what has been recently proposed by Nozari et al. (2011).

Using computational simulations and neuropsychological data, these authors provided a detailed theoretical account in which a domain-general conflict-detection mechanism, assumed to be hosted in the medial frontal lobe, plays a central role in the on-line monitoring of speech production by interacting with the speech production system itself. Their model was able to successfully predict error detection patterns of 29 aphasic patients performing a simple picture-naming task (i.e., the Philadelphia Naming Task, PNT). Although these authors do not deny the role of speech comprehension in speech monitoring, they suggest its role is limited to external feedback monitoring taking place after speech has actually been produced, as suggested by studies in which auditory feedback is either masked or distorted (e.g., Lackner and Tuller, 1979 and hemodynamic studies cited above). Inner speech monitoring could thus be subserved by interactions between medial frontal regions supporting a domain-general monitoring process and the speech production system itself. The data of one of the patients we recorded supports this idea that interactions between medial frontal regions generating the Ne and left temporal regions underlie inner speech monitoring. This patient had a lesion that was not limited to the LPFC but extended into the anterior left temporal lobe. Interestingly, this patient did not have a larger Ne in errors than in correct trials, mirroring the pattern reported outside of language in LPFC patients by Gehring and Knight (2000) and Ullsperger et al. (2002) and Ullsperger and von Cramon (2006).

### LIMITATIONS

One constraint is due to the small number of errors made by controls, which precluded measurement of reliable error components in controls and comparison of error and correct patterns between the patient and control groups. We note that both controls and patients had higher error rates than the young controls tested in Experiment 2 (also simple picture naming) of Riès et al. (2011), (average error rate: 1.31%, average number of errors: 16, SD = 12), and expected to be able to observe an Ne in both groups. Due to time recording constraints the total number of trials in both tasks was about half as in Experiment 2 of Riès et al. (2011), leading to too few errors for proper signal averaging in aged-matched controls for errors especially in the Simon task. Moreover, in our study more participants were in the low number-of-errors range than in Riès et al. (2011). In Riès et al. (2011), the nine participants kept for EEG signal averaging made nine errors or more. In the present experiment only eight participants made nine errors or more in the Naming task and only five made nine errors or more in the Simon task.

We also did not observe a clear Ne in correct trials in aged-matched controls in either task. No clear component was visible at FCz. The reason why aged-matched controls did not show a typical Ne in correct trials as reported in Riès et al. (2011) is not clear. The different number of trials could be one possible explanation. We note that Acheson et al. (2012), average error rate: 1.4%, reported in Christoffels et al., 2007b) recently reported an Ne in correct overt picture naming using overall less trials than in the present experiment, although the component they report peaked much later than the Ne reported here in patients and in young controls in Riès et al. (2011). An alternative explanation could be linked to the use of the possessive determiner “my” before the name of the picture. We asked participants to say “my” in front of the picture names to reduce jitter between vocal responses and because we recorded EMG activity of muscles involved in articulating labial phonemes. However, it is possible that this might have added some jitter in the averaging of monitoring-related activities. Indeed, as all utterances started with the same syllable, participants may have anticipated its articulation and could have started saying “my” before having fully planned the articulation of the name of the picture or of the rule-guided response in the Simon task. The fact we observed a clear Ne in correct and incorrect trials in patients suggests this jitter was not likely a major issue. Finally, the amplitude of the Ne in errors and in correct trials has been shown to be affected by difficulty in overt speech (Acheson et al., 2012) but also in linguistic tasks involving manual responses (Sebastian-Gallés et al., 2006; Ganushchak and Schiller, 2009) and in non-linguistic tasks (Gehring et al., 1993; Falkenstein et al., 2000; Allain et al., 2004): the Ne in correct trials is larger and the Ne in errors in smaller in more difficult situations. The fact we observed a Ne in correct trials in patients but not in aged-matched controls may be explained by the fact patients had more difficulty than controls in performing the task.

An additional limitation comes from the absence of difference in performance between left and right PFC patients. As mentioned in the introduction, damage to the left PFC has been associated to a range of language deficits, leading to the prediction that the left PFC patients would have more difficulty in performing the Naming task than the right PFC patients. This was however not the case. The number of patients in each group may have been too small for an effect of lesion side to emerge. Moreover, because of task requirements, the patients we kept for analysis had overall good language production and comprehension as assessed by standard neuropsychological testing which may have prevented observing differences between left and right PFC patients. Such a limitation may be hard to avoid in EEG studies of overt speech monitoring though possible differences between left and right PFC patients may be investigated using linguistic tasks involving manual responses.

## CONCLUSION

Taken together, our results shed new light on the network underlying on-line speech monitoring in simple overt picture naming. They reinforce the idea that a common node in the MFC is involved in speech and non-speech action monitoring manifested by the Ne potential. However, this domain-general monitoring system appears to be not as critically dependent on the LPFC in speech vs. other actions which involve arbitrary S-R mapping to be maintained in working memory. Moreover, in speech production, temporal regions are involved in monitoring not only through auditory feedback but also in on-line monitoring before speech is actually produced. This temporal involvement is particularly needed when lexical representations have to be accessed. We propose that a network of interacting brain regions involving medial frontal regions and temporal regions supports inner speech monitoring and that the role of the LPFC in action monitoring may be restricted to situations involving arbitrary stimulus-response associations to be maintained in working memory.

## Conflict of Interest Statement

The authors declare that the research was conducted in the absence of any commercial or financial relationships that could be construed as a potential conflict of interest.
